# Balancing risk and reward: Toward resilient human–primate coexistence in a rapidly changing environment in Sulawesi, Indonesia

**DOI:** 10.1007/s13280-026-02348-4

**Published:** 2026-02-12

**Authors:** Amanda L. Ellwanger, Kristen S. Morrow, Ashni Kumar Dhawale, Henry R. Scharf, Putu Oka Ngakan, Erin P. Riley

**Affiliations:** 1 Department of Anthropology, Georgia State University, 33 Gilmer Street, Atlanta, GA 30033 USA; 2 Department of Anthropology, University of Georgia, 355 S Jackson Street, Athens, GA 30602 USA; 3 Department of Anthropology, San Diego State University, 5500 Campanile Drive, San Diego, CA 92182 USA; 4 Department of Mathematics, University of Arizona, 617 N. Santa Rita Drive, Tucson, AZ 85721 USA; 5 Faculty of Forestry, Hasanuddin University, Jl. Sunu Kompleks Unhas B/9, Makassar, 90211 Indonesia

**Keywords:** Coadaptation, Coexistence, Human–primate interface, *Macaca maura*, Provisioning, Resilience

## Abstract

This study explores human–moor macaque (*Macaca maura*) coexistence in Sulawesi, Indonesia, using resilience thinking to assess temporal patterns of coadaptation amidst stressors such as provisioning and road construction. Comparing data from 2016–2017 to 2023–2025, we examine changes in provisioning patterns, macaque roadside use, and people’s perceptions of macaques to evaluate factors that may test the system’s resilience. Our results show that although provisioning frequency has remained stable, hand-feeding is increasingly common and macaques have increased their use of roadside habitat. Additionally, people’s perceptions have shifted from excitement and novelty to fear and normalization. Decreasing tolerance, coupled with increased risks associated with roadside behavior, highlights the system’s potential to transition to a state incompatible with coexistence. Our results can be leveraged to promote resilient coexistence, e.g., interventions that enable safer roadside crossing for the macaques and community outreach programs that make use of people’s empathy for the macaques’ welfare.

## Introduction

Across the globe, human–wildlife encounters are increasing in frequency as human populations grow and anthropogenic infrastructure and land use expands (Ma et al. 2024). Human–wildlife interactions can lead to conflict, defined as when humans and wildlife negatively impact each other (Madden 2004). Conflict has been a central narrative in human–wildlife studies, but coexistence is increasingly recognized as a valuable perspective to frame interactions (Frank et al. 2019; Bhatia et al. 2020; Pooley et al. 2021; Ellwanger 2025). Carter and Linnell (2016: 575) define coexistence as a dynamic but sustainable state in which humans and wildlife co-adapt to living in shared landscapes via effective behavioral management (human and animal) and risk tolerance. Human–wildlife coexistence does not exclude the possibility of conflict in the same system, as these are dynamic contexts where people and animals negotiate the benefits and risks of overlapping in space (Hill 2021).

Resilience theory is a useful framework to understand the socio-ecological processes underlying coexistence in human–wildlife systems (Carter and Linnell 2023). This framework models how much stress a system can absorb before experiencing a fundamental shift in structure or function, the adaptive capacity to change in response to such conditions, and the ability for systems to find a new stable state following change (Holling 1973; Gunderson 2000; Folke et al. 2010). A key element are thresholds, or tipping points, wherein a system experiences enough stress or perturbation that it shifts from one stable state to another (du Toit et al. 2004; Walker 2020). Leveraging resilience thinking in the context of human–wildlife coexistence, Carter and Linnell (2023) developed a heuristic model of eight archetypal relationships between people and wildlife, wherein each archetype is defined by the abilities of humans and animals to co-adapt to one another. These archetypes reflect a spectrum of coexistence, co-adaptation, and resilience, ranging from weak mutual adaptation, where conflict results from people and wildlife being unable to tolerate each other (e.g., “zero sum losers”), to strong mutual adaptation, where humans and wildlife mutually benefit from each other’s presence (e.g., “sustained co-benefits”). Importantly, human–wildlife systems may belong to more than one archetype at a time, moving between them depending on sociopolitical and/or environmental conditions. The applied significance of this heuristic model is to better understand how humans and wildlife adjust to perturbations, what factors lead to tipping points, and how to increase the resilience of states that are consistent with coexistence and decrease the resilience of those that are not.

**Fig. 1:**
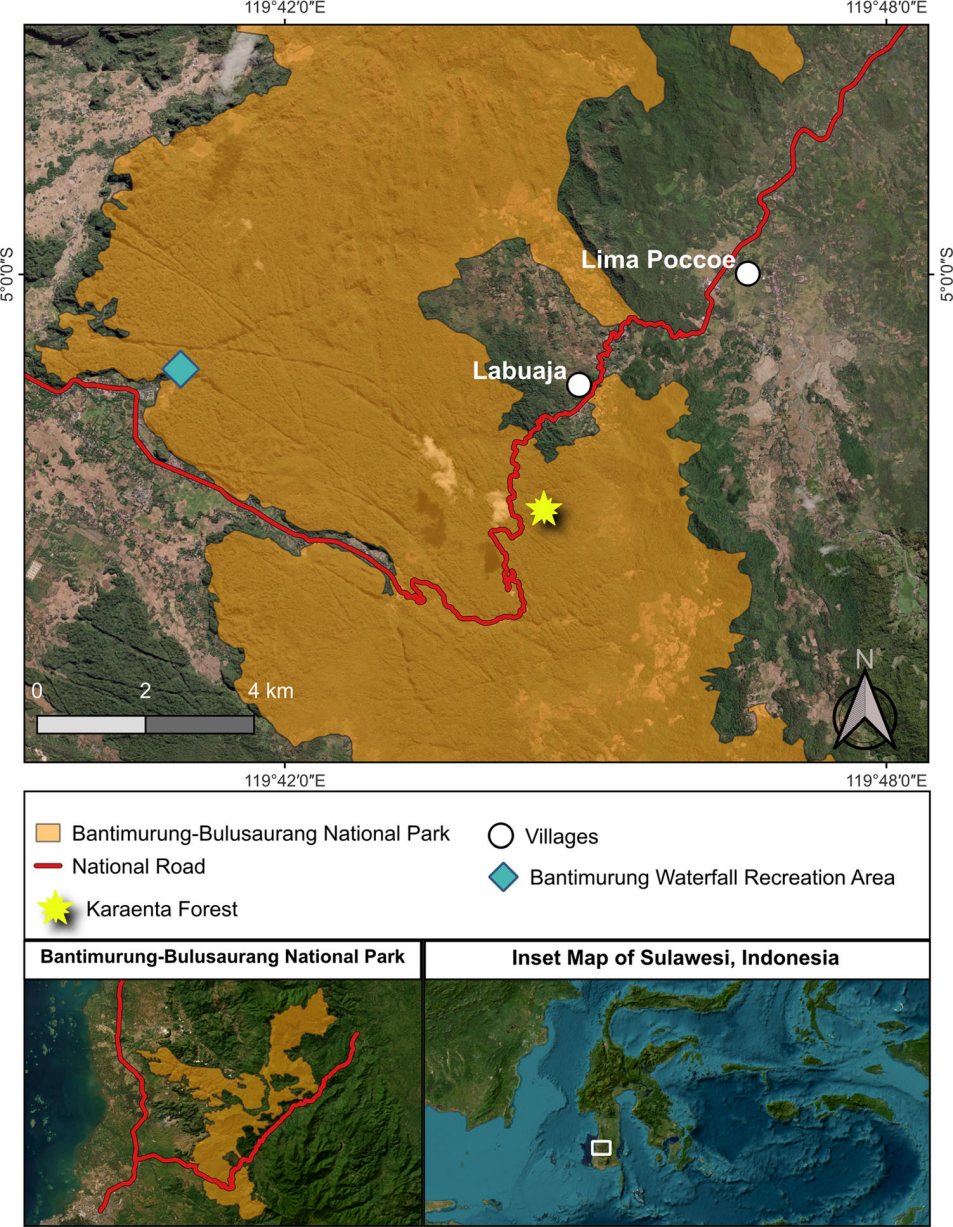
Map of study area depicting national park boundaries, national road, location of the Karaenta Forest, and locations where interviews were conducted

Here, we apply resilience thinking and Carter and Linnell’s (2023) “coadaptation archetypes” heuristic to examine an evolving interface between humans and moor macaques (*Macaca maura*) in Bantimurung–Bulusaraung National Park (TNBABUL) in Sulawesi, Indonesia and the potential for sustainable coexistence. The primate genus *Macaca* is well known for its behavioral flexibility and capacity to persist, and sometimes thrive, in human-dominated landscapes (Dhawale et al. 2020; Cooper et al. 2022). Sulawesi is recognized as a globally important conservation area because of its unique biodiversity and high levels of species endemism, coupled with the fact that its remaining forest cover is under threat as agricultural and mining sectors expand and human population size grows (Supriatna et al. 2020). Although TNBABUL is located > 3 km from human settlements, an economically important and heavily trafficked national road traverses through 11 km of the park (Fig. 1), bisecting the habitat of resident wildlife, including a population of Endangered moor macaques (Riley et al. 2020). In the past, resident macaque groups would cross the road, on the ground or in the canopy, and then retreat into the forest after crossing. Interactions with humans were limited to encounters with park staff, researchers, and occasionally local villagers who collect forest products, such as honey and palm sap to make palm sugar or juice/wine (*ballo*). Accordingly, at this site, moor macaques and people have historically lived in a state akin to Carter and Linnell’s (2023) “fragile stability.”

**Fig. 2:**
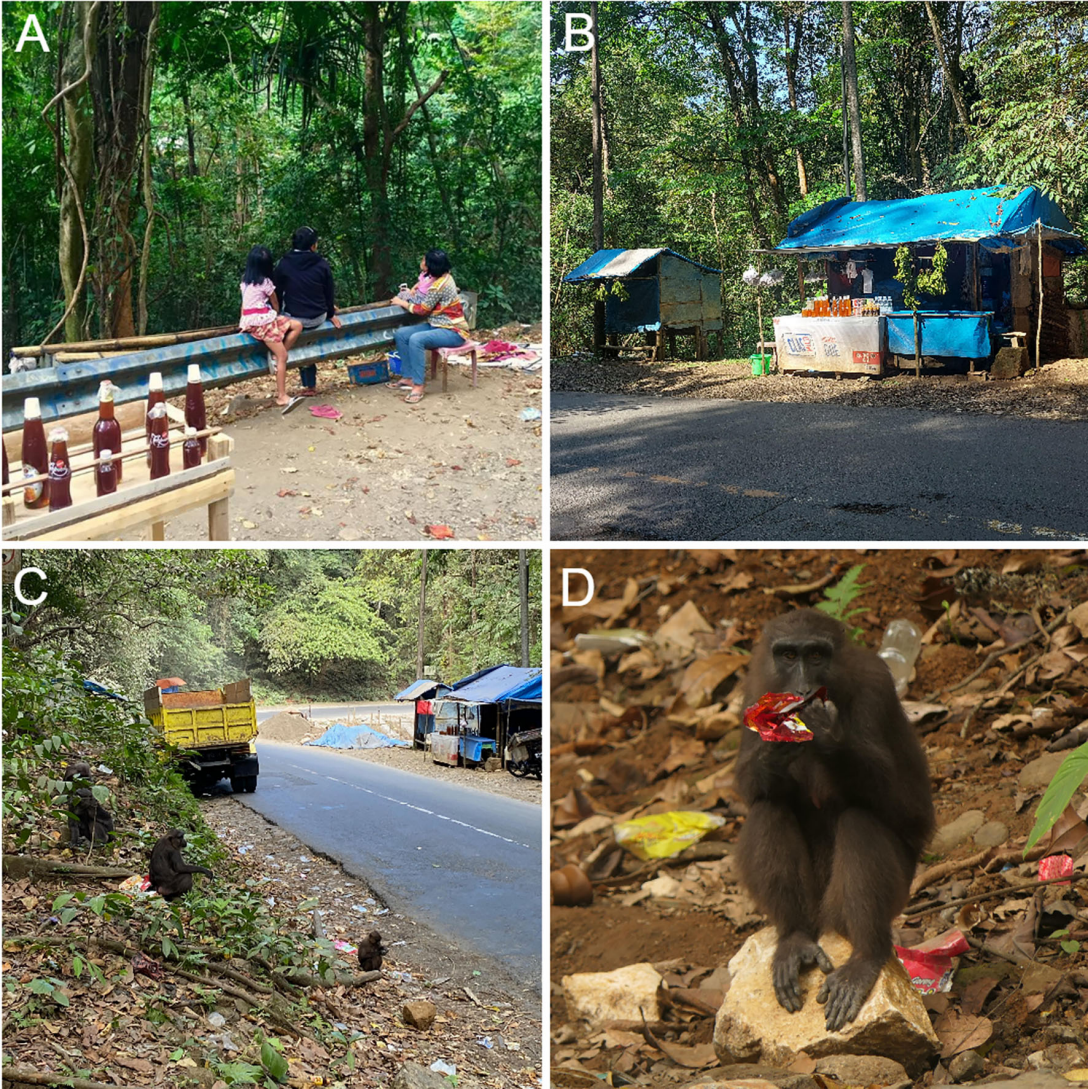
Roadside vendors in 2016–2017 (**A**) compared to larger, more formalized stalls present in 2023–2025 (**B**). Moor macaques forage in the litter that accumulates from vendor stalls and from passing motorists (**C**, **D**). Image credits: A: Kristen S. Morrow; B: Amanda L. Ellwanger; C. Amaru Marchant; D: Ashni K. Dhawale

**Fig. 3:**
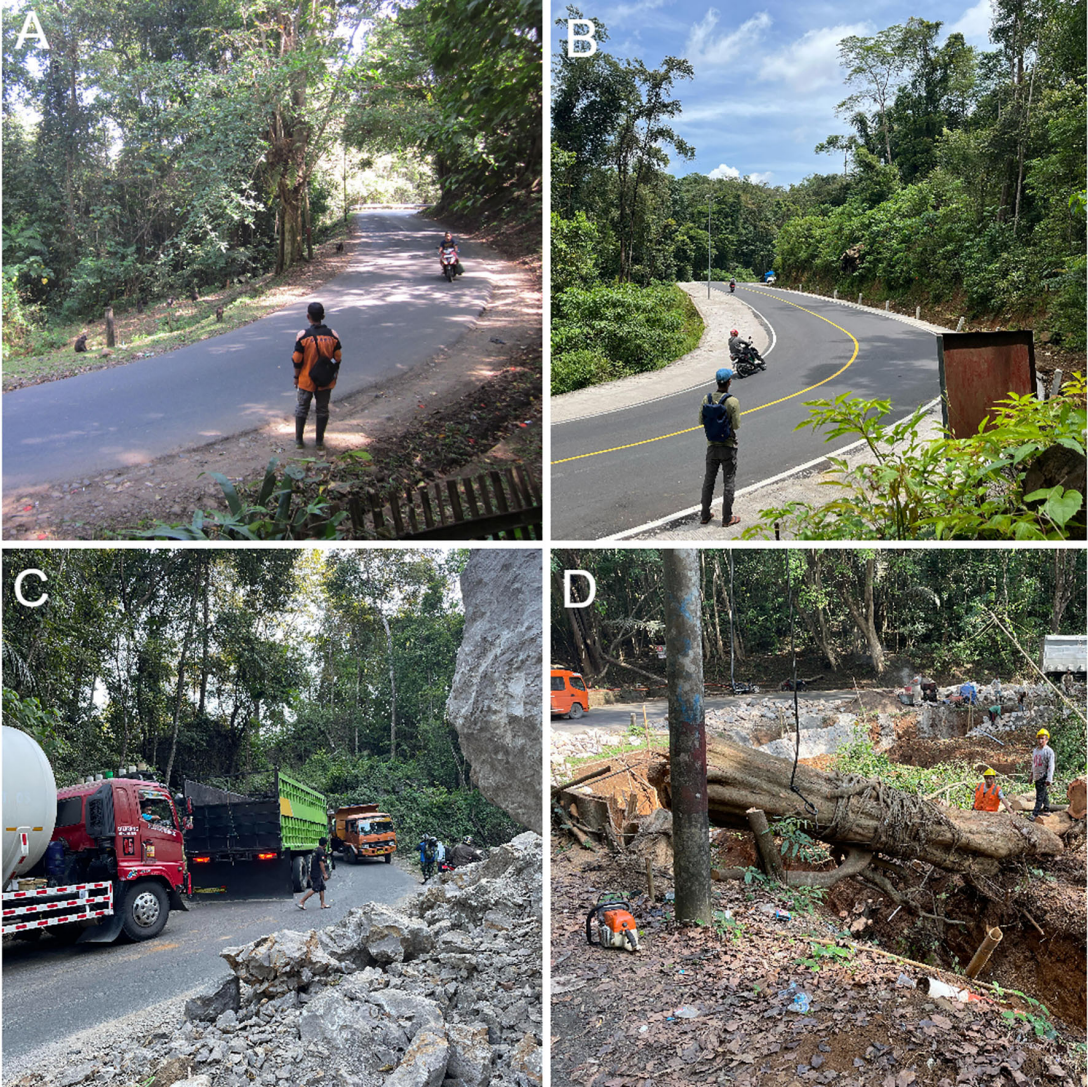
The national road in the park was more narrow in 2016–2017, with some canopy connectivity that facilitated arboreal road crossings by some macaques (**A**). By 2023, the road was wider and there was little to no arboreal connectivity and greater exposure to higher temperatures with less shade (**B**). Construction to expand the road began in 2022 and caused notable destruction of karst formations (**C**) and edge habitat (**D**). Image credits: A: Erin P. Riley; B: Ashni K. Dhawale; C: Erin P. Riley; D: Erin P. Riley

In 2015, this system experienced two major perturbations. First, a few residents from nearby villages began setting up small vendor stalls (*warungs*) along the road to sell forest products and other food items. These stalls meant the accumulation of food waste and other trash along the road as well as a greater presence of people (Fig. 2). Second, people passing in vehicles began provisioning one habituated group of macaques (Group B), which began spending more time along the road (Morrow et al. 2019). By 2018, we observed additional, unhabituated macaque groups waiting for provisions along the entire stretch of the road that passes through the park (Authors, unpubl.). We also documented a shift in Group B’s home range such that their core area encompassed more road and roadside habitat (Riley et al. 2021). In 2022, the system began experiencing a new perturbation: major ecological disturbances associated with a large-scale road-widening project, which involved the destruction of several meters of forest and karst formations on either side of the road (Fig. 3). By 2025, more than eight groups of macaques visit the roadside, vendors have grown in number to over 20, and provisioning from passing vehicles continues despite signage prohibiting it.

In this paper, our goal is twofold. First, to understand how people and moor macaques have co-adapted to their roadside interface over time. To do so, we compare patterns of macaque roadside habitat use, rates of roadside provisioning, and people’s perceptions and attitudes toward the macaques from 2016–2017 and 2023–2025. Provisioning contexts present a complex set of risks and rewards, such that we might expect certain age/sex classes (e.g., juveniles and adult females) to be more risk-averse and forego roadside habitat for safer forest interior habitat (Balasubramaniam et al. 2020). However, over time, individuals may learn how to adjust to risks associated with anthropogenic landscapes (e.g., roads; Tellier et al. 2025) and adjust their habitat use to reap potential rewards of being in proximity to humans (e.g., access to provisioned foods). We therefore predicted that there would be significant differences in roadside use across age-sex classes in 2016–2017, but that these differences would dissolve in 2024–2025 as individuals adapt to the changing context. We also predicted that the frequency of provisioning events would increase over time as people become more accustomed to encountering monkeys along the roadside and, hence are more prepared to feed them. We qualitatively analyzed interview data to identify potential shifts in people’s perceptions of macaques and motivations for interacting with macaques along the road. Second, we draw from our results to characterize past, present, and possible future archetypal states of this human-macaque interface and to generate recommendations for adaptive management strategies that could promote resilient coexistence.

## Materials and methods

### Research approach and context

We used an ethnoprimatological approach, which integrates primatological and ethnographic methods to examine interconnections between people and primates, and the implications for conservation and management (Riley and Ellwanger 2013; Dore et al. 2017). We conducted the research in the Karaenta forest area of Bantimurung–Bulusaraung National Park (TNBABUL) and two villages close to the national park: Labuaja (population: 2282 people) and Lima Poccoe (population: 3580 people) (BPS Maros Regency 2025) (Fig. 1). Karaenta is a 1000-ha section of TNBABUL characterized by mixed primary and secondary forest and seasonality in rainfall (typically, wet season: November–May; dry season: June–October). Comprising 43 750 ha, TNBABUL was gazetted in 2004 to protect the region’s karst ecosystem and biodiversity. Moor macaques live in cohesive multimale-multifemale groups, ranging in size from 15 to 40 individuals (Okamoto et al. 2000). They are primarily frugivorous, but also consume insects, leaves, and other vegetative material, and will feed on agricultural crops and provisioned foods (Zak and Riley 2017; Morrow et al. 2019; Albani et al. 2020).

### Behavioral data collection

Behavioral observations were conducted on one, well-habituated study group (Group B) in which all macaques were individually recognizable (Table 1). To understand patterns of macaque roadside use, we conducted group scans (Altmann 1974) at 30-min intervals. Each scan lasted 10 min during which one person recorded the location of each individual in Group B. Location was scored as ‘road’ (along the road or roadside areas lacking vegetation) or ‘forest’ (all locations further than 3 m from the roadside). From August 2016 to January 2017 data were collected on Group B six hours per day, six days per week, rotating morning (0600–1200 h) and afternoon (1200–1800 h) sampling periods, resulting in 1175 scan samples (21,063 behavioral records), with an average of 17.93 (± 6.40 SD) individuals observed per scan. From August 2024 to August 2025, data were collected on Group B from 0800 to 1700 h two days per week, resulting in 277 scan samples (2,809 behavioral records), with an average of 10.14 (± 4.63 SD) individuals observed per scan.

**Table 1:** Age-sex class distribution and total size of the moor macaque focal group across data collection periods, following categories defined by Okamoto et al. (2000) and Riley et al. (2016). Note: Due to a demographic shift, the juvenile females from 2016–2017 aged into different age/sex classes in 2024–2025

Age-sex class	2016–2017	2024–2025
Adult male (≥ 8)	9	8
Adult FEMALE (≥ 6 yrs)	7	6
Subadult Male (6–7 yrs)	2	4
Subadult female (6–5 yrs)	4	3
Juvenile male (1–5 yrs)	6	8
Juvenile female (1–4 yrs)	5	0
Infants (< 1)	2	3
Total	35	32

**Fig. 4:**
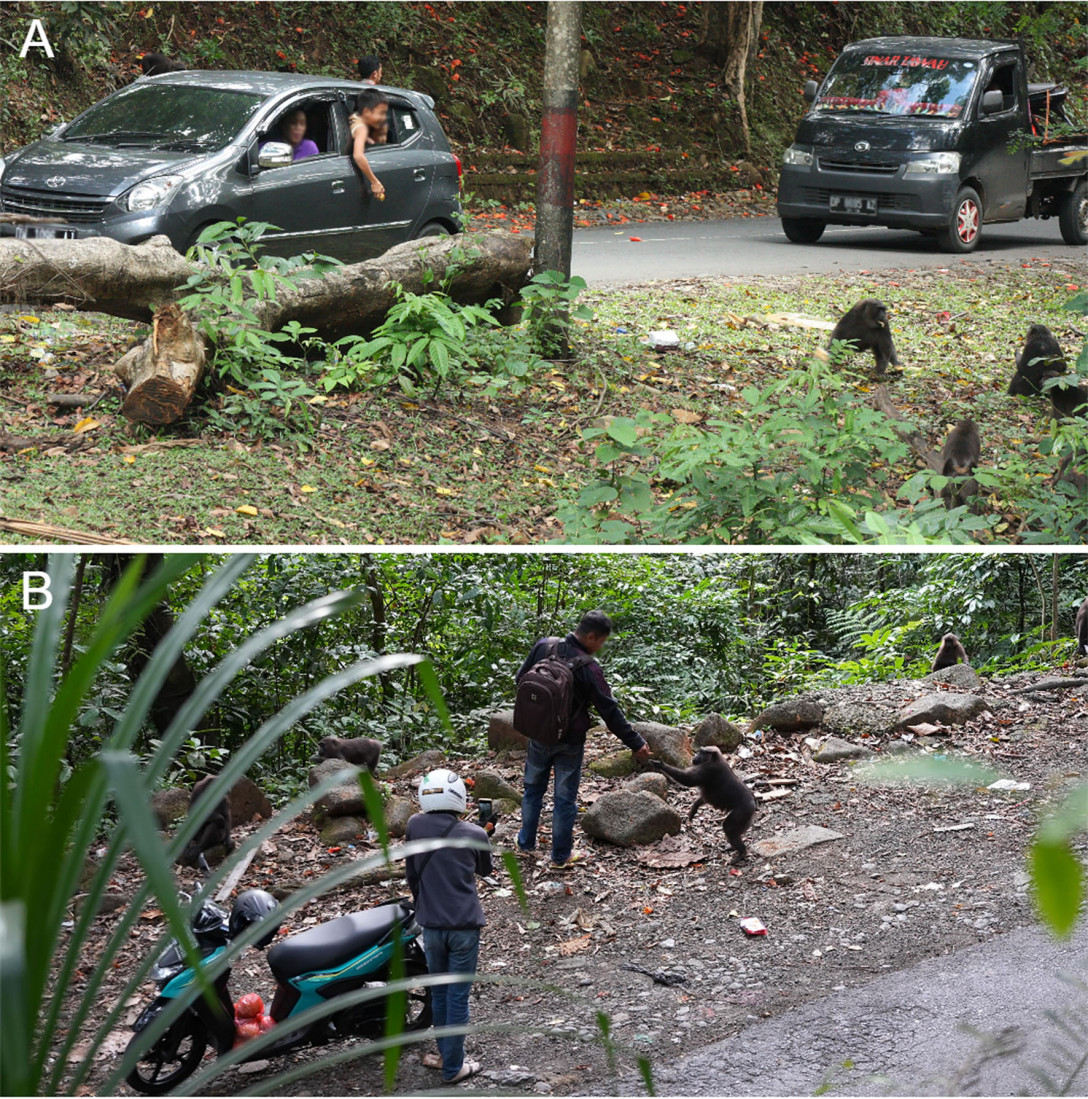
Examples of roadside provisioning of moor macaque monkeys in Bantimurung Bulusaraung National Park, Sulawesi, Indonesia. People will provision monkeys from their vehicles (**A**) and by tossing or handing food directly to the monkeys along the roadside (**B**). Image credits: A: Kristen S. Morrow; B: Elena Williams-Moreiras/Project SEED

We used all-occurrence sampling (Altmann 1974) to record provisioning events along the road directed toward either Group B or Group C, a neighboring group that we had begun habituating in August 2024. Following Riley and Wade (2016), 10-min all-occurrence samples were taken every 30 min, during which we recorded every observable provisioning event, along with the food type provisioned (when possible). We defined a provisioning event as any instance wherein macaques were provided food resources by people, whether from a moving vehicle, a stopped vehicle, or via hand-feeding (Fig. 4). Provisioning was recorded a maximum of one time for each vehicle. In 2016–2017, observations of provisioning were conducted six hours per day, six days per week from August to January, rotating morning (0600–1200 h) and afternoon (1200–1800 h) sampling periods. In 2024–2025, all-occurrence data were collected one day per week between 0800-1700 h from August 2024 to August 2025. We recorded 284 all-occurrence samples from 2016–2017 and 198 all-occurrence samples from 2024–2025.

### Ethnographic data collection

Across the study periods, we conducted semi-structured interviews with 73 adult (≥ 18 years old) respondents recruited using convenience and purposive sampling (Bernard 2018) (Table 2). This framework allowed us to conduct in-depth interviews with respondents who have direct knowledge and experience of the macaque’s behaviors and interactions with people. Respondents included national park staff, residents of Labuaja and Lima Poccoe, and visitors to the national park. We conducted interviews at roadside vendor stalls (*warungs*), the waterfall recreation site, and in the villages due to proximity to the macaque provisioning sites. Two of 19 village residents interviewed in 2016–2017 and 20 of 42 interviewed in 2023–2024 worked as vendors at *warungs* inside the park. Interviews were conducted in Bahasa Indonesia, with the help of an Indonesian research assistant when necessary. Interviews consisted of sociodemographic and open-ended questions, which were used to understand people’s knowledge of macaque feeding ecology, perceptions of the monkeys, and motivations for provisioning. Interview protocols were similar across time periods, enabling our team to create comparative datasets, even though we observed and interviewed different people. Respondent demographics shifted between study periods due to sampling focus and an increase in the number of *warungs* (i.e., a greater focus on park employees in 2016–2017, who are mostly men, and *warungs* in 2023–2024, which are largely run by women).

**Table 2:** Demographics of respondents interviewed across study periods. The occupation category “other” includes a variety of roles including drivers, household business, homemakers, students, and government employees

	2016–2017	2023–2024
Age	19–64 (37.8 average)	20–78 (42.4 average)
Sex	7 women 14 men	34 women 18 men
Ethnicity	Bugis, Makassar- 19 Toraja- 1 Sasak- 1	Bugis, Makassar- 51 Javanese- 1
Occupation	Farmers- 0 Park employee- 5 Forest *warung* vendor- 2 Other- 14	Farmer- 8 Park employee- 0 Forest *warung* vendor- 20 Other- 24

### Data analysis

#### Macaque roadside use

**Fig. 5:**
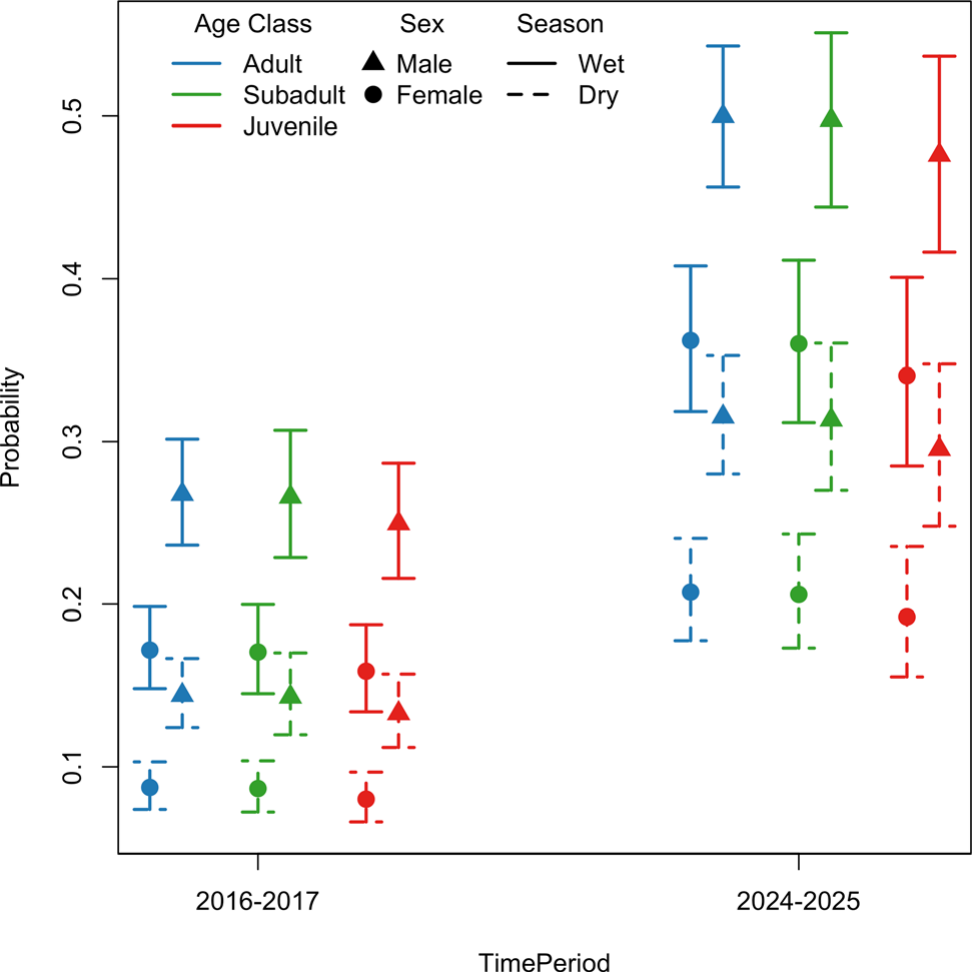
Predicted probabilities (95% confidence intervals based on an additive GLMM) of observing an individual from different age and sex classes near the road during either wet or dry season in the two study periods

We calculated the percentage of scans containing at least one macaque in the roadside habitat and the percentage of individual scan records in roadside habitat for each study period. We also fit generalized linear mixed models (binomial GLMMs with logit link function; Agresti 2013) to examine factors influencing the probability of observing moor macaques in the roadside habitat (0 = forest and 1 = road). Factors considered included Sex (male, female), Age Class (juvenile, subadult, adult) and Time Period (2016–2017 vs. 2024–2025) as fixed effects, and a random intercept for Individual to account for repeated measures. To account for seasonal differences in sampling across the study periods, we also included Season as a binary fixed effect (wet vs. dry). To select an appropriate model for road use, an initial GLMM with all fixed factors, the interactions of Time Period with Age Class, Sex, and Season, and a random effect for Individual were considered and then simplified using a stepwise procedure. All interaction terms were iteratively dropped yielding a final, additive model including Time Period, Age Class, Sex, Season, and random effect for Individual. Fewer scan sampling and all-occurrence sampling days were devoted to Group B in the 2024–2025 study period for two reasons: the other days of the week were devoted to data collection for other research objectives, including expanding our sampling effort to include observations of an additional macaque group (Group C). Nonetheless, one benefit of using a GLMM for data analysis is that the model inherently accounts for the difference in the number of samples (and hence behavioral records) between the two time periods, yielding trustworthy inference despite the imbalance of records (see, e.g. wider confidence intervals for 2024–25 in Fig. 5). Computation and model fitting were performed using R (v4.4.2; R Core Team 2024) using the *lme4* package (v1.1–37; Bates et al. 2015).

#### Provisioning

To examine whether the frequency of provisioning differed between time periods, we used a Chi-squared test comparing the distributions of counts of provisioning events during all-occurrence samples in each time period (2016–2017 and 2024–2025) in R using base packages. To investigate whether rates of hand-feeding differed between time periods, we used a Chi-squared test comparing counts of hand-feeding and all other provisioning methods across time periods. To determine most commonly provisioned food types, we organized provisioned foods into the following categories: cultivated food (e.g., fruits, vegetables, and legumes), packaged and cooked food (e.g., bread, chips, cookies, rice, noodles, tofu), trash, beverages, or unknown. We then calculated the percent contribution of each of these categories across all provisioning events for each study period. While there was a moderate difference in all-occurrence sample sizes between time periods, the Chi-squared tests used in our analysis account for unequal samples in the calculation of associated statistics and *p* values.

#### Perceptions of macaques and motivations for interacting

Interview data were transcribed, translated, and qualitatively analyzed through open coding and grounded theory to identify emergent themes related to perceptions of macaques and people’s experiences and motivations for interacting with or provisioning the monkeys (Ryan and Bernard 2003). We coded open-ended data by hand and using NVivo 15 and compared emergent themes across study periods. Four major themes emerged: the prominence of cultivated and packaged food types; increasing fear of the monkeys; empathy as a driver for provisioning; and decreasing novelty of encountering monkeys along the roadside.

## Results

### Macaque roadside use increased across time periods, with males being the most frequent users

In 2016–2017, 29% of scans contained at least one record of an individual in the roadside habitat; in 2024–2025, 44% of scans recorded at least one individual in the roadside habitat. Additionally, 17% of individual scan records were recorded in roadside habitat in 2016–2017, compared to 32% of individual scan records in 2024–2025.

The GLMM revealed significant influences of Time Period and Season on the probability of observing macaques in the roadside habitat (Table 3; Fig. 5). Namely, macaques in Group B used the roadside habitat significantly more often in 2024–2025 (vs. 2016–2017) and during the wet season in both time periods. In contrast to our prediction, age class was not a significant predictor of roadside habitat use in either period (Table 3). However, in partial support of our prediction, sex was a significant predictor of roadside use, with males being more likely than females to be observed along the roadside in both periods (Table 3). These results suggest that females in Group B have remained more risk averse relative to males, despite extended exposure to the roadside context.

**Table 3:** Additive effects of time period, age class, and sex on the probability of an individual being observed near the road

Factor	Coefficient	*p*-value
Time Period (2024–25 vs. 2016–17)	1.007 ± 0.073 SE	< 0.001
Age Class (Subadult vs. Adult)	− 0.016 ± 0.098 SE	0.871
Age Class (Juvenile vs. Adult)	− 0.094 ± 0.111 SE	0.398
Sex (Male vs. Female)	0.564 ± 0.099 SE	< 0.001
Season (Wet vs. Dry)	0.774 ± 0.037 SE	< 0.001

### Frequency of provisioning is similar across time periods, but hand-feeding has increased over time

At least one instance of provisioning was observed in 42% of the all-occurrence samples from 2016–2017 and in 47% of the samples from 2024–2025. In contrast to our prediction, we did not find a significant difference in counts of provisioning events per sample between 2016–2017 and 2024–2025 (*χ*^2^ = 7.5669**,** *p* = 0.371). Although hand-feeding was an uncommon provisioning method in both time periods, we observed a significant change in the proportion of hand-feeding provisioning events from 2.7% in 2016–2017 to 7.3% in 2024–2025 (*χ*^2^ = 4.359, *p* = 0.0443).

### People predominantly provision packaged foods and cultivated fruits

**Fig. 6:**
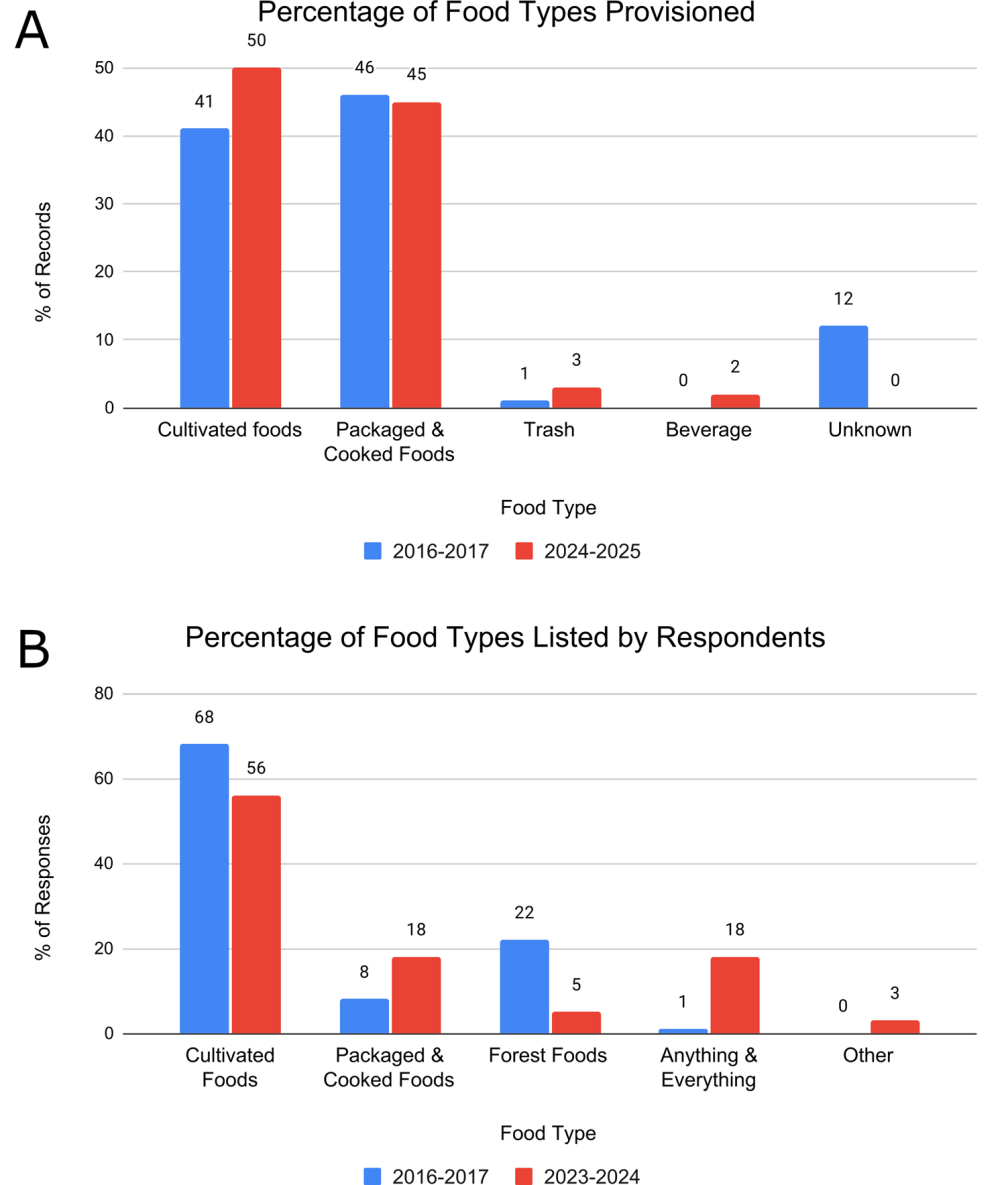
Percentage of each food type provisioned to macaques during observed provisioning events (**A**) and percentage of each food type that interview respondents listed in response to the prompt, “What do macaques like to eat?” (**B**)

Packaged and cooked foods were provisioned at similar proportions across study periods, though the proportion of cultivated foods provisioned increased in 2024–2025 (Fig. 6). Bananas were the most commonly provisioned cultivated food in both time periods (78.5% of cultivated foods in 2016–2017 and 77.3% in 2024–2025), although people also provisioned mango, papaya, jackfruit, watermelon, corn, peanuts, guava, oranges, and sweet potato. Breads, cakes, chips, rice, noodles, and *krupuk* (deep fried crackers) were commonly provisioned packaged and cooked foods (46% in 2016–2017 and 45% in 2024–2025).

### People perceive cultivated fruit to be primary macaque foods, but say the monkeys will eat almost anything

When asked “What do macaque monkeys like to eat?” over half of respondents in both time periods listed cultivated foods (68% in 2016–2017 and 56% in 2024–2025) (Fig. 6), and bananas were listed most frequently out of all cultivated items. In 2016–2017, forest foods (i.e., insects, leaves, and fruits) were the next most common food type listed. Packaged and cooked foods comprised only 8% of responses and only one response was coded as “anything and everything.” In contrast, in 2023–2024, the second and third largest categories were packaged foods and “anything and everything,” which were described as “anything people give them,” “anything you throw,” or “everything people eat.” Forest foods accounted for a smaller percentage of responses in 2023–2024 (5%) compared to 2016–2017 (22%).

### Over time more people reported being fearful of roadside monkeys

In 2016–2017, 67% respondents described roadside macaques as tame (*jinak*) rather than wild (*liar*) because “they always hope for food from humans” (man, age 28), the macaques allowed people to approach closely, or they were not fearful of cars. Additionally, 38% of respondents in 2016–2017 believed the macaques encountered on the road were under human care (*dipelihara*) and reliant on people for food. A third of the respondents compared roadside macaques to “wild” macaques seen around farms and gardens, which were characterized as naughty (*nakal*) but smart (*pintar*) animals that strategically arrive when people are not present to consume crops. In contrast, roadside monkeys were described by 67% of respondents in positive terms (good, cute, funny, and sweet). In 2023–2024, these differences were less clear cut. Respondents noted that roadside monkeys had gone from being friendly and tame to “wild, but comfortable” (man, age 50) and naughty. Whereas in the past the macaques could be chased away, they are no longer afraid of people and respondents associated this transformation with provisioning. As one respondent commented, “[In Karaenta] they’re not afraid of humans because we give them food. The monkeys in Karaenta are naughty. But here [in the village], if the monkeys see humans, they go back into the forest” (man, age 45). Out of 40 respondents who reside in the nearby villages, including vendors, 12.5% noted this change in behavior. In 2023–2024, over half of respondents (59%) reported that they were afraid of the monkeys and viewed them as dangerous, aggressive, unpredictable, or wild, compared to only 14% of respondents in 2016–2017. No respondents in 2016–2017 reported actually having been threatened by the monkeys, and only 10% (two respondents) heard of others being threatened. In contrast, in 2023–2024, 14% (seven respondents) reported being threatened by monkeys—primarily in the context of food.

### Empathy accounts for why people provision macaques

In both study periods, respondents expressed empathy for roadside macaques (43% of respondents in 2016–2017 and 29% of respondents in 2023–2024) and this served as a driver for provisioning. Respondents reported feeling sorry for the macaques (*kasihan*) and perceived them to be hungry and waiting for food. As one respondent stated in 2017, “It’s humane [to feed them]. I also get hungry, just like animals…Us humans are the same, so we give them food because we feel compassion” (woman, age 39). In both periods, half of respondents (56% of non-park employees (*N* = 16) in 2016–2017 and 49% of respondents in 2023–2024) reported having provisioned the monkeys.

### The novelty of encountering monkeys has waned over time

In 2016–2017, approximately half of respondents (48%) expressed that seeing macaques along the road was new (*baru*) and 57% expressed curiosity (*tertarik*), excitement (*gembira*), or happiness (*senang*) at seeing them; in 2023–2024, just 18% of respondents expressed similar views. In 2016–2017, people described encounters with roadside macaques as novel and unique, and some reported prolonging interactions to observe or photograph the monkeys by slowly feeding them small pieces of food. In contrast, over half (55%) of respondents in 2023–2024 expressed neutral feelings and reported that seeing the monkey was “just normal” (*biasa saja*), compared to 24% of respondents expressing similar views in 2016–2017. Almost a third of respondents (30%) in 2023–2024 expressed negative attitudes wherein respondents remarked that they would throw rocks or chase the monkeys away if they saw them. Negative emotions were associated with fear of being threatened, attacked, or simply in proximity to the monkeys, as well as concern for damage to crops or vendor products. In 2016–2017, only national park staff (*n* = 5) expressed negative attitudes, primarily oriented around managing roadside safety.

## Discussion

In the contemporary era in which human–wildlife interactions are increasingly common, it is critical to assess how species co-adapt to each other and which factors influence the resilience of coexistence. Here we integrated resilience thinking and ethnoprimatology to examine the changing human–macaque interface and the potential for coexistence in Sulawesi, Indonesia.

### Macaques are balancing risk and reward at the roadside

One of our objectives was to examine how macaques have behaviorally adapted to an expanding road infrastructure and increased opportunities to interact with people via provisioning along the roadside. Provisioning can result in overselection of anthropogenic habitat and a decrease in consumption of native foods (Sengupta and Radhakrishna 2018; Hansen et al. 2020). In this study, we found that Group B has increased the amount of time spent along the roadside. This finding indicates that the roadside continues to present opportunities for the macaques to gain quick access to calorie-rich foods, and they are shifting their foraging patterns to exploit these opportunities. That said, in both time periods, the majority of scan records were in the forest, indicating that they still rely on forest habitat and resources. This result is promising in that the macaques continue to access native foods that provide a balance of nutrients needed for growth and reproduction (Felton et al. 2009). Continued use of forest habitat also means that the macaques can continue providing ecological services, such as seed dispersal (Sengupta et al. 2015). However, increased roadside use is not unique to our main study group (Group B), as the number of groups that use the roadside has increased in the last 10 years from one in 2015 to at least eight in 2025. Whether the behavior has spread via dispersing males influencing their new groups or novel invention is a question that remains to be studied. Future research should explore at what threshold would a continued increase in roadside habitat use begin to negatively impact the macaques’ ecological roles as seed dispersers and the resilience of the ecological community.

Provisioning contexts can be risky for primates, particularly along roadsides due to the potential to be injured or killed by vehicles (Riley et al. 2021; Ilham 2024). In contrast to our prediction, we did not find a significant difference in roadside habitat use between more risk averse (i.e., juvenile) and less risk averse (i.e., adult) age classes in either time period. This could be explained by other life history factors of the juvenile period, such as higher levels of curiosity and a propensity toward novel behaviors and social learning (Barrett et al. 2017; Perry 2020). We did, however, find a significant sex difference in the probability of being on the road, with females from all age classes being less likely than males in both time periods. This suggests that females’ perception of risk has remained consistent over time. That said, we continue to observe some adult females with infants in very close proximity to the road and, in October 2024, an infant was struck and killed by a passing motorcycle. Accordingly, moor macaques at our site, and wildlife who use roadside habitat more broadly, must balance the risks and rewards that roads present, which points to a certain level of fragility in the state of coexistence.

### Provisioning primates: Shifting methods and perceptions

When food provisioning of wildlife first emerges in a new context, it often rapidly grows in popularity, leading to dramatic changes in population size (e.g., Japanese macaques; Kurita 2014). Contrary to our prediction, the frequency of provisioning remained similar across time periods. This is likely due to the practical difficulty of stopping along the road, particularly during road construction, and suggests that in roadside or similar contexts (i.e., where people are largely transient or space is restricted) there are limits on the extent to which provisioning can expand. The mismatch between what people feed the macaques (i.e., packaged foods) and what people think the monkeys prefer eating (i.e., cultivated foods) suggests that people are provisioning the monkeys opportunistically and simply sharing whatever they have at the time. This is in contrast to other provisioning sites where people come prepared with food specifically for wildlife (e.g., rhesus macaques at Silver River, Florida; Riley and Wade 2016). Moreover, opportunistic provisioning with snack foods has likely reinforced people’s observations that the monkeys will eat anything and everything, lending support for the growing co-adaptation of people and macaques to each other’s presence.

Although the frequency of provisioning has not increased significantly over time, we found that provisioning methods shifted, with hand-feeding being increasingly common. Directly handing food to primates is inherently risky for people and primates, as it increases the likelihood of being bit or scratched, and hence, the likelihood of zoonotic infection (e.g., rabies and/or Simian Herpes B Virus; Kennedy et al. 2019). Hand-feeding likewise exposes macaques to pathogens of human origin via saliva (e.g., tourists cracking peanut shells with their mouths before feeding Barbary macaques in Morocco; Maréchal et al. 2016) or respiratory pathways. Regardless of these risks, efforts to be in close proximity and interact with wildlife are often motivated by a desire for fun, memorable experiences, a sense of empathy, and a way to connect with nature (Sengupta and Radhakrishna 2020; Waters et al. 2025) and our interview results indicate the same. Therefore, like the macaques, people are experiencing a risk—reward tradeoff in this system.

People’s perceptions of the monkeys, however, are shifting. Rather than classifying animals as strictly wild or domestic, the terms *jinak* (tame) and *liar* (wild) allow animals to be classified on a spectrum based on their reactions to or reliance on people. For instance, a dog that is fearful of humans may be considered *liar* until it is friendly with people, at which point it is considered *jinak*. Our study group has a long history of habituation, a mutually modifying process whereby primates and people get used to the presence of one another (Hanson and Riley 2018), thereby muddying the waters between wild and tame (Birke 2014). Because our main study group was not fearful of people when they initially began using roadside habitat, their “tameness” encouraged people to feed and interact with them. Now, more respondents described them as *nakal* (naughty) and *pintar* (smart), words previously applied to crop foraging monkeys around farms (Zak 2016). People are spending more time along the roadside, as the number of *warungs* has grown, and are now recognizing more commonalities between the macaques who live around the farm and macaques who live in Karaenta.

Increased interspecies aggression has been well documented in provisioning contexts where respondents report that monkeys initiate aggressive encounters, but it is often stimulated by people’s reaction to the threat of economic loss (Beisner et al. 2015). In addition to potential injuries, interspecies aggression is associated with elevated stress and anxiety in macaques (Maréchal et al. 2011). At our site, people and macaques have become habituated to provisioning, resulting in perceptions that the monkeys have grown more aggressive. This perception is not unfounded, as in recent years we have observed individual monkeys boldly approach moving vehicles or motorcycles and aggressively swipe or slap at them, for example. Although our sampling framework makes it difficult to generalize to a wider population, our results do capture the experiences of people in this context on a daily basis. Moreover, while recall bias may reduce respondent accuracy (i.e., are the monkeys *actually* more aggressive now than they were 10 years ago?), it does not invalidate people’s perceptions, which form the basis of their beliefs and behavioral responses to perceived conflict (Dickman 2010).

### Implications for the state of human-macaque coexistence

**Fig. 7:**
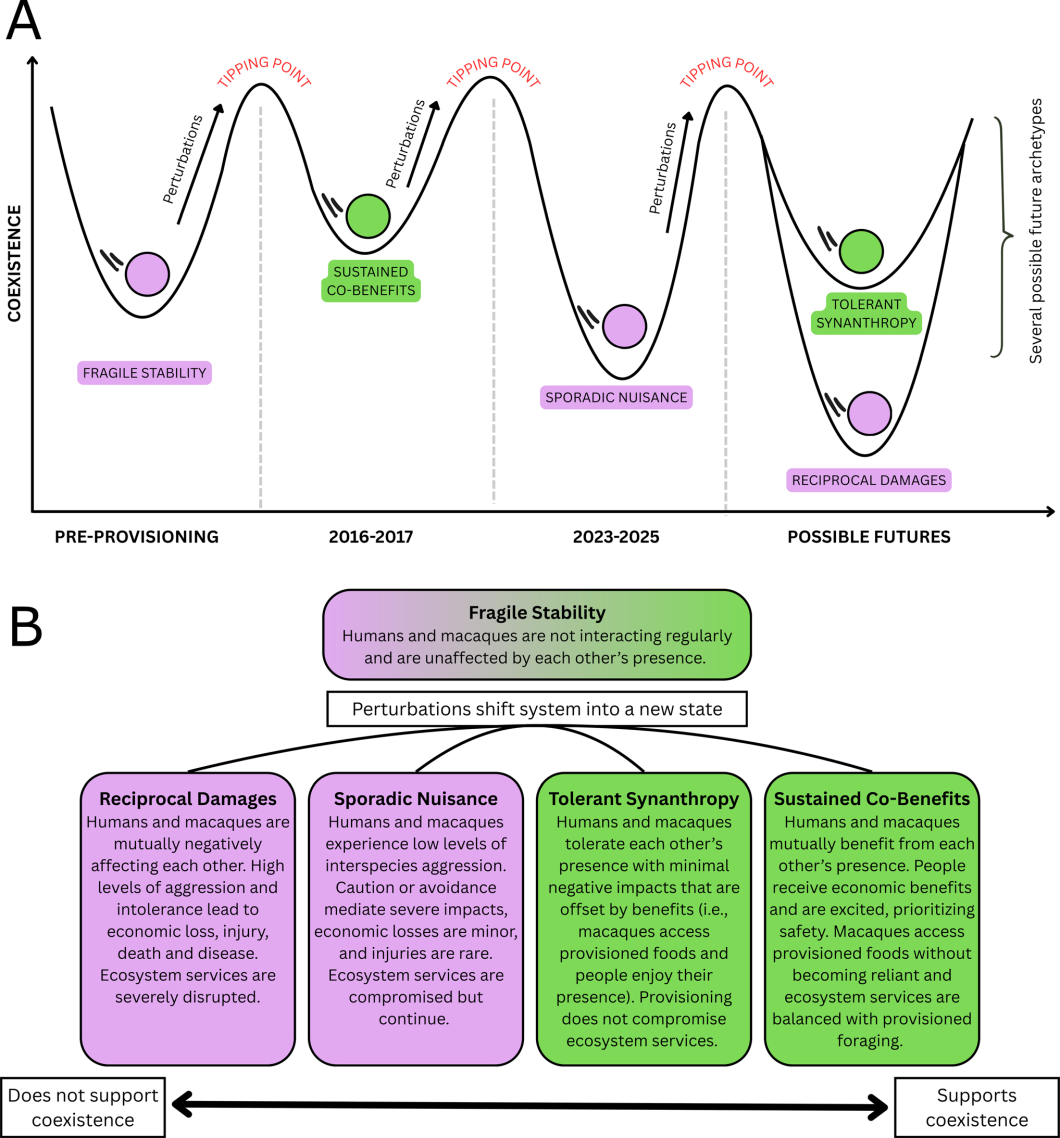
Various perturbations have shifted the archetypes at this site. (**A**) Following the emergence of provisioning, and novel encounters with macaques along the roadside, the system shifted from “fragile stability” to sustained “co-benefits”. Subsequently, daily provisioning, regular roadside use by the monkeys, increasing numbers of vendors stalls along the road, an ecologically destructive road expansion project, and waning tolerance for the monkeys shifts the system to “sporadic nuisance”. Future archetypes could move toward or away from coexistence. (**B**) Descriptions of past, present, and possible future archetypes in our system over time. Archetypes are positioned along a spectrum from least supportive of coexistence (green) to most supportive of coexistence (purple)

The Karaenta forest in TNBABUL, which is home to moor macaques and provides passageway for people, has experienced multiple perturbations over the past 10 years including the introduction and expansion of roadside *warungs*, provisioning from people in passing vehicles, and a major road widening project. Our data indicate that, in 2015, the emergence of provisioning and vendor stalls along the road likely shifted the system from a state of “fragile stability” to one more consistent with coexistence, “sustained co-benefits” (Fig. 7). People and macaques mutually benefited from these interactions, with provisioned foods providing additional nutritional sources for the monkeys, vendors tolerating the macaques near *warungs*, and people expressing content over encountering the monkeys. By 2023, subsequent perturbations, including road construction, increasing numbers of *warungs*, and an increased amount of time spent along the roadside by macaques, may have further shifted the system to a state less consistent with coexistence: “sporadic nuisance.” Increasing roadside use has meant greater potential costs, including increased likelihood of vehicle collisions, increased exposure to zoonoses, thermal constraints from being on the roadside, and an increased likelihood of negative interactions with people, especially given the increase in hand-feeding. People now perceive the macaques as more aggressive and no longer see encounters as novel and exciting. These changes indicate that tolerance of the monkeys and perceptions of derived benefits may be waning.

What do these results mean for the future of the system? Although disturbance in ecological systems is often viewed as driving population decline (e.g., drought, disease, or fragmentation: Strier 2021), access to denser caloric foods can increase reproductive rates and support population growth, which would be desirable for an Endangered species like the moor macaque, reflecting strong demographic resilience (Capdevila et al. 2020; Cao et al. 2025). However, diets heavy in provisioned foods can have negative health consequences for wildlife, including micro- and macronutrient deficiencies, altered immune function, exposure to contaminants, elevated cholesterol, and obesity (Murray et al. 2016; Rothman and Bryer 2019). While we have not yet examined the nutritional impacts of provisioning, there is evidence that the group’s dietary diversity is reduced while foraging along the road, compared with inside the forest (Williams-Moreiras et al., in preparation). In addition, since 2024, several individuals from two of our study groups (Groups B and C) have exhibited skin lesions and hair loss, possibly reflecting allergic reactions and/or infection (Authors, unpubl.). Further research is needed to determine the health consequences of provisioning, in terms of likelihood of infections, allergic reactions, and the macaques’ ability to balance their nutritional needs. A broader issue concerns overall ecosystem health, as provisioning may weaken ecological resilience by disrupting ecosystem services if fewer seeds are dispersed or dispersed in areas less favorable for germination (i.e., closer to parent tree, on road surface; Sengupta et al. 2015). Accordingly, provisioning may represent a trade-off between demographic resilience for the macaques and ecological resilience for the ecosystem.

Continued research at this site will allow us to better understand how the changing dynamics of the human-macaque interface may further shift the system closer to (e.g., tolerant synanthropy) or away from (e.g., reciprocal damages) coexistence (Fig. 7). While people are motivated to feed and interact with roadside macaques in Karaenta, our findings reveal that the same species encountered in farmlands a few kilometers away are conceptualized—and responded to—much differently. These differences reinforce the notion that coexistence occurs along a spectrum, and the same species may fall under different archetypes of coexistence or conflict even when in proximate geographic spaces (Radhakrishna et al. 2025).

## Conclusions and management implications

Our comparison of ethological and ethnographic data collected across two time periods provides evidence that people and moor macaques are co-adapting to one another in the face of perturbations related to socioeconomic, sociocultural, and environmental factors. These findings underscore macaques’ capacity for behavioral and ecological flexibility in response to anthropogenic influence (Hansen et al. 2020; Cooper et al. 2022), which may be a key factor promoting resilience (Gladstone-Gallagher et al. 2019). Furthermore, our data indicate that people’s perceptions of wildlife may be more malleable than animal or human behaviors, highlighting the relational nature of human–wildlife coexistence and showing how human-primate interfaces can shift to “sporadic nuisance” or “reciprocal damages” if primates become more aggressive toward people (e.g., Govindrajan 2015).

We acknowledge that the behavioral data we report come from a single group of macaques, which limits the generalizability of our findings. However, a preliminary analysis of our behavioral sampling effort for a neighboring group, Group C, that we recently habituated to observer presence, indicates that we found the group along the road 50% of the days devoted to searching for them (*n* = 108). Future comparative analysis of their roadside behavior with that of Group B will expand our understanding of the role the history and extent of habituation to humans plays in shaping primate behavior and driving behavioral flexibility. Our findings can be scaled up to inform future research, strategies to achieve human–wildlife coexistence, and conservation management plans more broadly. For example, given the variation we found between male and female macaques in roadside use, future studies could examine how other individual level factors, such as personality, facilitate or weaken coexistence (Gladstone-Gallagher et al. 2019; Capdevila et al. 2020). In terms of conservation management, future efforts could explore ways to further facilitate the macaques’ behavioral adaptation to the roadside dynamic, such as installing artificial canopy bridges that enable them to cross the road more safely (Yap et al. 2022).

Our findings also demonstrate that people’s conceptualizations of wildlife are shaped by the risks they face when encountering them, which in turn influence the extent to which people may tolerate—or be intolerant of—encounters. In contexts such as this, conservation management of human–wildlife interactions should prioritize minimizing people’s risk of injury or damage when encountering wildlife, as doing so may reduce negative perceptions of them and increase tolerance and resilience of synanthropy. For instance, providing information to roadside vendors on how to best store their goods may prevent economic loss from macaques and encouraging better waste management along the roadside could decrease the attractiveness of this habitat for the macaques (Teampanpong 2021; IUCN SSC Primate Specialist Group 2025).

Previous research has shown that people’s behavior toward primates often elicits unwanted responses (McCarthy et al. 2009; Beisner et al. 2015) and that people, in turn, often misinterpret primate behavior and emotional states (Maréchal et al. 2017). Educational programs instituted in local communities could provide guidance on how to observe the macaques without potentially eliciting aggression (e.g., avoiding pointing or approaching quickly, correctly interpreting macaque body language/expressions). Outreach programs should leverage our finding that empathy is a major driver of provisioning at our site. That provisioning continues despite numerous signs clearly stating that the practice is prohibited suggests that signage alone is not a strong enough intervention (Dickman 2010). Provisioning is a culturally-situated act that is not intrinsically detrimental or unnatural (Waters et al. 2025). Re-focusing the messaging of outreach programs toward notions of care, such as emphasizing negative consequences of provisioning (e.g., risk of vehicle collision; getting sick from eating human foods) or at minimum discouraging the provisioning of foods that are potentially harmful to macaques (e.g., processed foods) may be more effective. In the last two years, we collaboratively developed and piloted an educational outreach program called “*Hati-Hati, Monyet!*” (“*Be careful, Monkey!*” in Bahasa Indonesia) for elementary-aged students that focuses on increasing knowledge and awareness of moor macaques and promoting safe human-monkey interactions. In the future, we will expand this program and incorporate formalized assessment to ascertain its efficacy. Recognizing provisioning as a culturally-situated act embraces a reflexive approach to the management, which will lead to more socially accepted and effective management strategies (Boyce et al. 2021), potentially increasing the resilience of human-macaque coexistence at this site.

Understanding what encourages people to be tolerant of wildlife may be key to applying resilience thinking to managing human–wildlife coexistence. Resilience thinking can be challenging to apply to wildlife management due to the unpredictable nature of human–wildlife systems and the potential difficulty of categorizing such nuanced systems into just one archetype. However, when used as a way to define the scope of a problem, assess stability or fragility of coexistence, and identify perturbations and potential tipping points, resilience thinking can help guide conservation management by helping to set objectives, identify how systems function, and encourage conservationists to anticipate a range of outcomes that may result from management actions (Fischer et al. 2009; Johnson et al. 2013).

## Data Availability

The data that support the findings of this study are available upon reasonable request.
